# BI-5756 Reduces Graft-Versus-Host Disease Through CB1-Mediated Treg Upregulation

**DOI:** 10.3390/molecules30173517

**Published:** 2025-08-28

**Authors:** Sena Kim, Abdul-Jalil Dania, Sora Lim, Jaebok Choi

**Affiliations:** Division of Oncology, Department of Medicine, Washington University School of Medicine, Saint Louis, MO 63110, USA; senakim@wustl.edu (S.K.); a.d.dania@wustl.edu (A.-J.D.); slim22@wustl.edu (S.L.)

**Keywords:** graft-versus-host disease, cannabinoid receptor1, regulatory T cells

## Abstract

Cannabinoid receptor 1 (CB1) has been implicated in multiple inflammatory diseases by regulating pro-inflammatory mediators or altering immune cell polarization. However, the expression and direct functional role of CB1 in T cells remain largely unexplored. Here, we demonstrate that primary murine T cells express CB1 and that its novel agonist, BI-5756, directly increases the frequencies of regulatory T cells (Tregs) in primary murine pan T cells after activation. In addition, BI-5756 exhibits an in vivo protective effect against graft-versus-host disease (GvHD), an allogeneic T cell-mediated inflammatory complication after allogeneic hematopoietic cell transplantation (allo-HCT), resulting in an improved overall survival with enhanced platelet recovery and reconstitution of bone marrow-derived B and T cells. BI-5756 also directly suppresses tumor cell growth and upregulates MHC I, MHC II, and CD80 on tumor cells, which may subsequently enhance T cell-mediated anti-tumor responses in mixed lymphocyte reaction with A20 cells. The ability of BI-5756 to increase Tregs was significantly abrogated by rimonabant, a potent and selective CB1 antagonist, suggesting that the immunomodulatory effect of BI-5756 is mediated via CB1. In summary, BI-5756, a potent CB1 agonist, increases Tregs while preserving anti-tumor responses in vitro and effectively reduces GvHD in vivo.

## 1. Introduction

Allogenic cell transplantation (allo-HCT) is a curative therapy for hematological malignancies that primarily relies on the graft-versus-leukemia (GvL) effect, in which donor T cells eliminate recipient residual malignant cells and prevent relapse. However, graft-versus-host disease (GvHD) arises from the same alloreactive T cells responsible for GvL. GvHD is allogeneic T cell-mediated damage of healthy recipient tissues and organs [1]. The development of therapies that can ameliorate GvHD while maintaining the GvL effect is critical for long-term GvHD-/relapse-free survival after allo-HCT.

Regulatory T cells (Tregs) are a specialized subpopulation of CD4 T cells, distinguished by a high expression of CD25 and the transcription factor FOXP3. Through the suppression of the proliferation and effector function of other immune cells, Tregs regulate the immune responses and maintain homeostasis and self-tolerance [2,3]. A recent phase II clinical trial evaluated Treg therapy in patients with myeloid malignancies undergoing myeloablative conditioning [4]. The administration of donor-derived Tregs 2–3 days prior to infusion of donor conventional T cells resulted in a dramatically low 1-year incidence of grade III-IV acute GvHD (only 7%) and improved immune reconstitution. Moreover, GvHD-/relapse-free survival (GRFS) at 1 year was 64%, which is significantly above the 40% estimated historical incidence of GRFS. While utilizing Tregs has been demonstrated as one of the most promising cellular therapies to prevent GvHD without compromising the GvL effect in both human patients and murine models after allo-HCT [5,6,7,8], their clinical application remains limited due to major challenges in generating sufficient numbers of Tregs and maintaining their suppressor functions ex vivo, as Tregs are a very rare population in peripheral blood, constituting approximately 10% in CD4 T cells and unstable in maintaining the expression of genes associated with immunosuppressive functions [9,10]. Therefore, identifying alternative methods that promote Treg expansion and suppressor functions, as well as understanding the mechanisms by which cell signaling modulates Treg differentiation and proliferation, may provide invaluable insights into advancing Treg therapy.

The endocannabinoid system (ECS) is a biological network of lipid-based neurotransmitters (cannabinoids) and their receptors (cannabinoid receptors), which regulate key physiological functions, such as pain, satiety, fear, memory, and immune responses [11]. Endocannabinoids are endogenous signal ligands for cannabinoid receptors (CBs), and structurally similar phytochemicals from the cannabis plant can also activate CBs. Cannabinoid receptor 1 (CB1) and cannabinoid receptor 2 (CB2) are the main CBs and have been reported to modulate the function of the immune system in various models of inflammatory diseases [12]. Cannabinoids, such as Δ9-Tetrahydrocannabinol (a psychotropic) and cannabidiol (nonpsychotropic), i.e., ingredients found in cannabis, have shown anti-inflammatory and immunosuppressive properties in mouse models of GvHD and clinical trials, primarily through CB1, CB2, and non-cannabinoid receptors [13,14].

GvHD arises as a result of donor-derived T cells recognizing recipient tissues as foreign, leading to uncontrolled T cell activation, expansion, and subsequent tissue damage. Therefore, suppressing GvHD-inducing T cell responses by reducing antigen presentation via MHC classes I and II, co-stimulatory signals such as CD80, and pro-inflammatory cytokines while promoting the expansion of GvHD-suppressing Tregs is a promising strategy to mitigate GvHD. As CB2 is highly abundant on immune cells, including T cells, and involved in cell proliferation and survival [15,16], it has been studied relatively more than CB1 for its protective effects in GvHD. In contrast, CB1, predominantly expressed in neurons of the central nervous system, is known to mediate psychoactivity rather than immunomodulatory functions [17,18]. Consequently, the functional roles of CB1 in immune cells are rarely investigated due to CB1 expression on immune cells being much lower than that of CB2 [11]. Only ACEA has been studied in T cells as a CB1 agonist, demonstrating impaired T cell proliferation induced by anti-CD3 monoclonal antibody in human peripheral blood mononuclear cells (PBMCs), whereas the CB1 antagonist SR141716A reversed this effect [19]. ACEA also significantly downregulated a T cell activation marker, CD25, on CD4 and CD8 T cells. Unlike the CB2 agonist GW833972A, ACEA does not suppress the cytotoxic activities of CD8 T cells [19]. Similarly, another CB1 antagonist, rimonabant, impaired the capacity of human monocyte-derived tolerogenic DCs to prime allogeneic naïve T cells, thereby eventually reducing the generation of Tregs in a co-culture experiment [20]. In addition, hepatic mononuclear cells (HMNCs) from db/db mice revealed significantly decreased frequencies of Tregs compared to lean control mice, which was reversed by administering AM251, a CB1 antagonist [21].

Currently, several CB1 agonists and antagonists have been developed [22,23,24,25]. They have a wide range of potential in modulating immune function depending on their chemical structure, but studies focused on T cells with new CB1 agonists have rarely been conducted. Although BI-5756 was originally designed as a cholesteryl ester transfer protein (CETP) inhibitor to increase high-density lipoprotein-cholesterol (HDL-C) while reducing low-density lipoprotein-cholesterol (LDL-C), rodents lack the CETP gene. Given its potential clinical use as a CETP inhibitor, we investigated potential primary off-target effects of BI-5756 in our mouse model of GvHD. BI-5756 exhibited a half-maximal inhibitory concentration (IC50) of 0.966 μM for CB1 in a cell-based radioligand binding assay using CP55940, a synthetic cannabinoid (SafetyScreen44^TM^), and increased the functional receptor activation by 58% at 10 μM and by 34% at 1 μM compared to the control in a GPCR GTPgamma assay, demonstrating that BI-5756 is a potent CB1 agonist [26].

CB1 agonists may represent a promising therapeutic avenue for immune modulation through their effect on the frequency and function of Tregs. To date, only ACEA has been evaluated in T cells, showing inhibitory effects on T cell proliferation and activation while sparing cytotoxic activities of CD8 T cells against tumors. In this study, we report, for the first time, that BI-5756 functions as a CB1 agonist and immune modulator of T cells. In addition, we evaluate the effect of BI-5756 on T cell-mediated GvHD in a preclinical mouse model of allo-HCT. Taken together, BI-5756 may provide therapeutic benefits not only as a CETP inhibitor but also through its additional off-target CB1 agonist activity, offering a novel mechanism for reducing GvHD.

## 2. Results

### 2.1. BI-5756 Upregulates Regulatory T Cells

**Figure 1:**
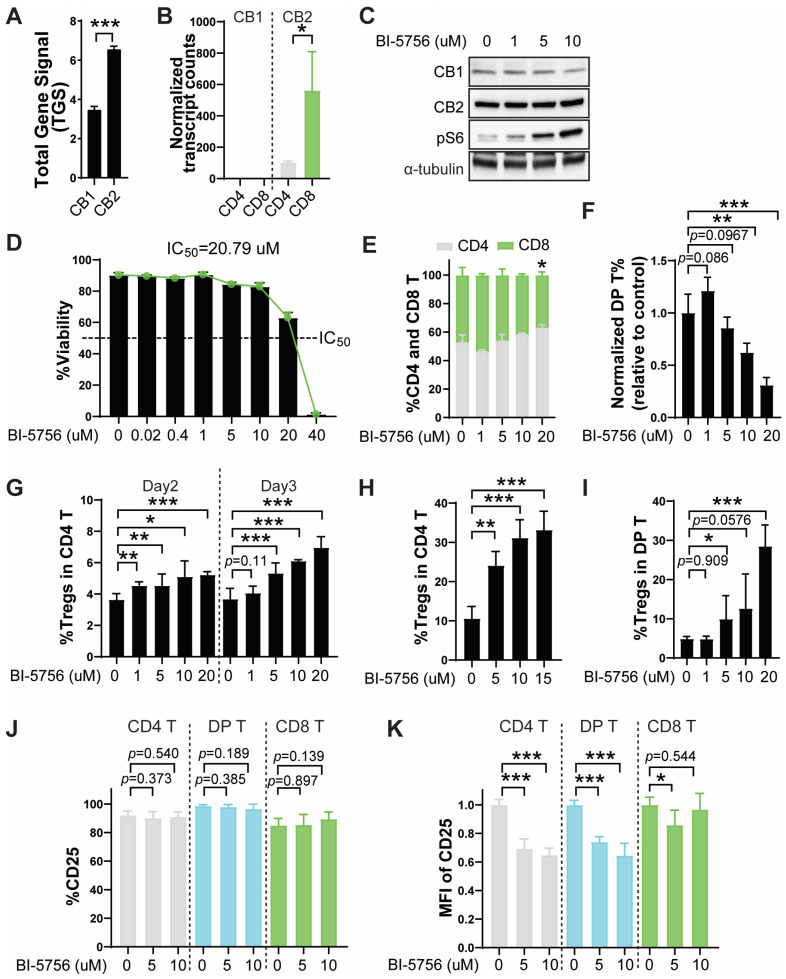
BI-5756 upregulates regulatory T cells in primary murine pan T cell cultures. (**A**) Total gene signal (TGS) of CB1 and CB2 in activated CD4 T cells on day 6 after MLR, generated by Affymetrix Mouse Gene 2.0 ST Arrays. (**B**) Normalized transcript counts of CB1 and CB2 from mRNA sequencing of resting T cells. (**C**) Protein expressions of CB1, CB2, and pS6 in pan T cells stimulated with anti-CD3/CD28 activation beads for 3 days, as determined by Western blotting; α-tubulin was used as a loading control. (**D**) The %viability of T cells after 3 days of activation in the presence or absence of BI-5756 at the indicated concentrations. T cells were stimulated with anti-CD3/CD28 activation beads for 2 days (**E**) and/or 3 days (**F**,**G**,**I**–**K**) or with allogeneic APCs for 6 days (**G**). (**E**–**I**) The CD4, CD8, and CD4 and CD8 DP T cells, as well as Tregs, were determined by flow cytometry. Tregs were identified by GFP-positive cells within the CD4 T cell population. (**J**,**K**) The %CD25-expressing T cells and mean fluorescence intensity (MFI) of CD25 in T cells were determined by flow cytometry. * *p* < 0.05, ** *p* < 0.01, and *** *p* < 0.001. All error bars are represented as mean ± standard deviation.

Since no studies have reported CB1 protein expression in primary murine T cells, we first examined the expression of CB1 and CB2 in T cells stimulated with anti-CD3/CD28 activation beads. Consistent with our mRNA expression data (Figure 1A,B) and reports from other groups showing that the expression of CB1 is lower than that of CB2 in human immune cells, TCR-activated primary T cells expressed both CB1 and CB2 at the protein level, with CB1 lower than CB2, as determined by Western blotting (Figure 1C). Although BI-5756 did not affect the expression of CB1 and CB2, it activated mTOR signaling, as shown by a dose-dependent increase in phosphorylated S6 (pS6), a well-established CB1 and CB2 downstream molecule in various cell types (Figure 1C). Next, we assessed whether the synthetic CB1 agonist BI-5756 could modulate T cell phenotype and function. BI-5756 was well-tolerated and showed no toxicity at indicated concentrations below 20 μM, with a half-maximal inhibitory concentration (IC50) of 20.79 μM (Figure 1D). At these concentrations, BI-5756 did not alter the ratio of CD4 and CD8 T cell frequencies, although a slight increase in the proportion of CD4 T cells was observed at 20 μM or higher in primary murine pan T cells obtained from Foxp3-ires-GFP knock-in mice and stimulated with either anti-CD3/CD28 activation beads (Figure 1E). Interestingly, BI-5756 dose-dependently decreased the frequencies of CD4 and CD8 double-positive (DP) cells, which are known to be highly potent inducers of GvHD by recognizing antigens presented on both major histocompatibility complex (MHC) classes I and II (Figure 1F). In addition, BI-5756 promoted the frequencies of FOXP3/GFP-positive Tregs in primary murine pan T cells stimulated with either anti-CD3/CD28 activation beads or allogeneic antigen-presenting cells (APCs) in a dose-dependent manner (Figure 1G,H). BI-5756 not only suppressed the frequencies of DP T cells but also increased FOXP3/GFP-positive Tregs within the DP T cell population, suggesting that BI-5756 reduces highly potent GvHD-inducing DP T cells (Figure 1F) while increasing GvHD-suppressing Tregs (Figure 1I). These data suggest that BI-5756 reduces the activation of CD4 and DP T cells, accompanied by a significant decrease in *Ifng* expression (Supplementary Figure S1), which is associated with Th1 differentiation while increasing Tregs, thereby significantly decreasing the Th1/Treg ratio favorable for GvHD protection. Other cytokines, including *Tgfb*, *Il6*, *Tnfa*, *Il1a*, *Il10*, and *Il4*, were rarely expressed and not affected by BI-5756 treatment (Supplementary Figure S1). To further support the CB1-specific effects of BI-5756, we examined an additional CB1 agonist, CB1 agonist 1 (pIC50 = 5.7), which similarly increased Treg frequencies in a dose-dependent manner in activated primary murine T cells (Supplementary Figure S2). Although BI-5756 did not reduce the frequencies of CD25-expressing T cells (Figure 1J), it selectively downregulated CD25 expression levels in CD4 and DP T cells (Figure 1K). In contrast, CD25 expression in CD8 T cells was less affected by BI-5756, indicating that the drug preserves CD8 T cell activation (Figure 1K). Since BI-5756 dose-dependently increased Tregs without significant toxicity below 20 μM, 10 μM was selected as the optimal effective non-toxic dose for subsequent experiments. These results suggest that BI-5756 does not affect T cell proliferation or viability but significantly upregulates Tregs and selectively suppresses CD4 and DP T cells by downregulating CD25 expression while preserving CD8 T cell activation, which potentially contributes to maintaining T cell-mediated anti-tumor response.

### 2.2. The Effect of BI-5756 on GvHD in a Preclinical Mouse Model of Allo-HCT

**Figure 2:**
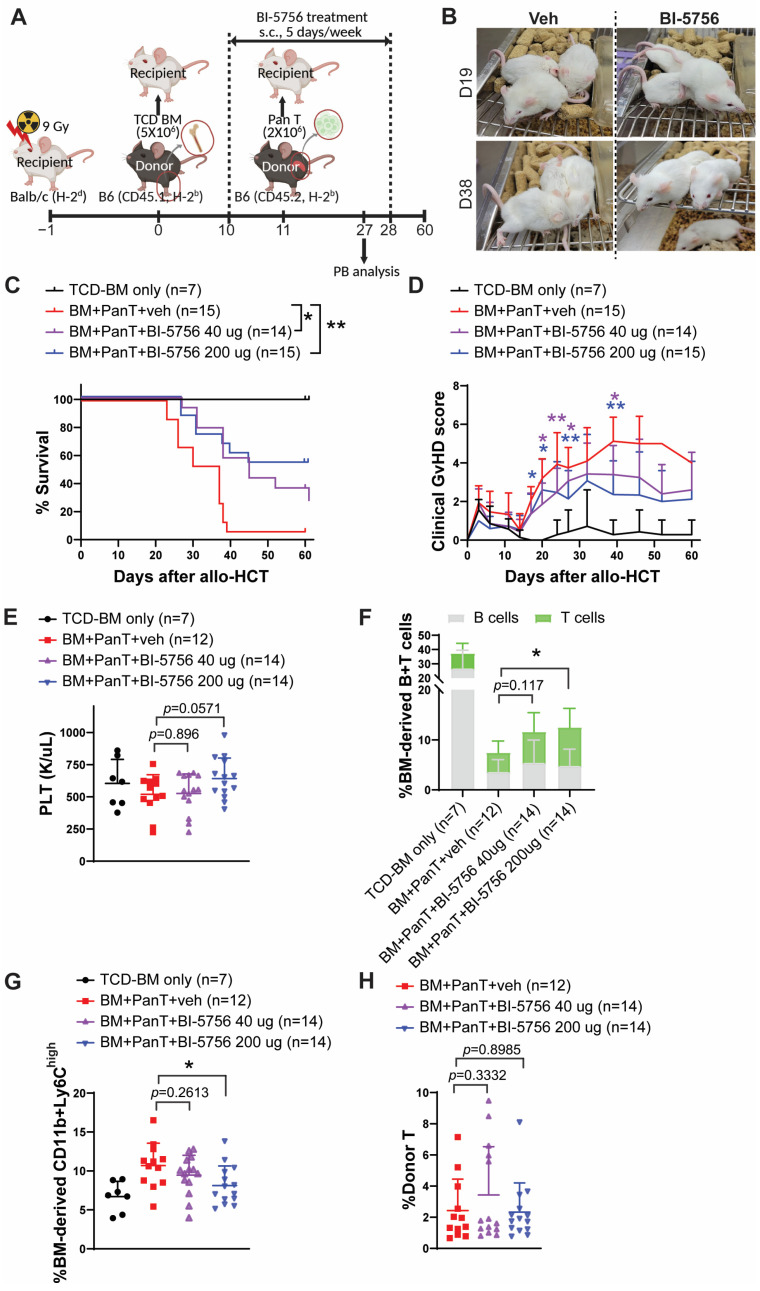
The effect of BI-5756 on GvHD in a preclinical mouse model of allo-HCT. (**A**) Schema of the mouse experimental design and BI-5756 treatment schedule in the allo-HCT mouse model. Created with BioRender.com (accessed on 27 August 2025). (**B**) Representative images of mice administered with vehicle control or BI-5756 (40 µg) on day 19 or day 38 after allo-HCT. (**C**) Survival rate and (**D**) clinical GvHD scores of mice after allo-HCT. (**E**) Blood cell count for platelets (PLTs) using whole blood on day 27 after allo-HCT. (**F**–**H**) Peripheral blood was analyzed for % donor BM-derived B cells and T cells (**F**), % donor BM-derived CD11b+Ly6C^high^ cells (**G**), and % donor T cells on day 27 after allo-HCT. A pool of two independent experiments is shown. * *p* < 0.05 and ** *p* < 0.01. All error bars are represented as mean ± standard deviation.

Tregs have potent immunosuppressive functions to protect and maintain immune homeostasis in various inflammatory diseases. The generation and maintenance of Tregs are indispensable for reducing the risk of GvHD and enhancing immune cell reconstitution after allo-HCT. Given our observation that BI-5756 upregulates Tregs (Figure 1), we evaluated its in vivo therapeutic efficacy in reducing GvHD in a preclinical mouse model of allo-HCT. Recipient BALB/c mice were lethally irradiated (900 cGy) one day before transplantation with T cell-depleted bone marrow (TCD-BM) obtained from C57BL/6 mice (CD45.1+). Eleven days later, delayed lymphocyte infusion (DLI) was performed by administering allogeneic T cells obtained from C57BL/6 mice (CD45.2+). In vivo administration of BI-5756 was started one day before DLI, 5 days per week, subcutaneously at 40 µg or 200 µg per injection per mouse for 3 weeks (Figure 2A). BI-5756 significantly improved overall survival with less clinical GvHD compared to vehicle control (Figure 2B–D). In addition, mice treated with BI-5756 exhibited a trend toward improved platelet (PLT) count recovery (Figure 2E) and significantly increased frequencies of donor BM-derived B cells and T cells (Figure 2F) in peripheral blood on day 27 after allo-HCT compared to those treated with vehicle control, which indicates less GvHD and improved donor BM immune reconstitution. Of note, the frequencies of BM-derived CD11b+Ly6C^high^ cells were reduced in mice treated with BI-5756 compared to those with vehicle control (Figure 2G). CD11b+Ly6C^high^ cells, primarily classical monocytes and neutrophils, are considered GvHD-inducing cell types due to their ability to present alloantigens to donor T cells and produce pro-inflammatory cytokines. However, frequencies of donor T cells were comparable between groups regardless of BI-5756 treatment on day 27 in peripheral blood after allo-HCT (Figure 2H). These results suggest that BI-5756 is a promising novel CB1 agonist with therapeutic potential to reduce GvHD in a preclinical mouse model of allo-HCT.

### 2.3. BI-5756 Directly Inhibits Tumor Cell Growth and Enhances Antigen Presentation in Murine B Cell Lymphoma Cells, A20

**Figure 3:**
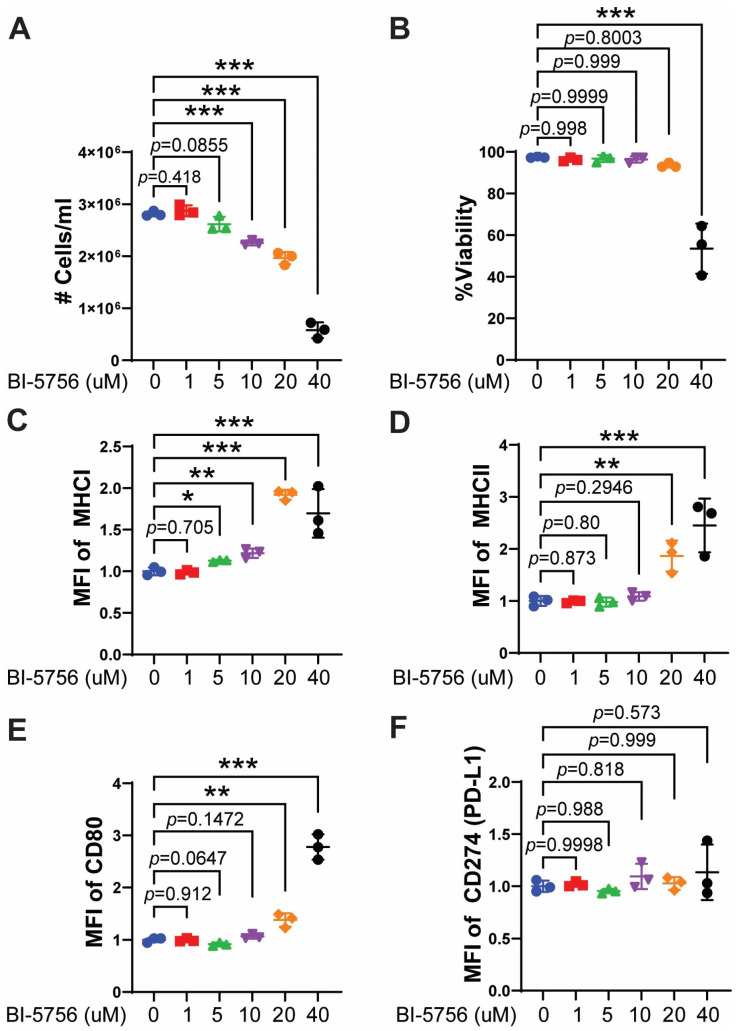
BI-5756 directly inhibits tumor cell growth and enhances antigen presentation in murine B cell lymphoma cells, A20. BALB/c-derived A20 tumor cells were treated with BI-5756 at the indicated concentrations for 24 hrs. (**A**) Viable cell numbers (#) per ml and (**B**) %viability were determined by acridine orange/propidium iodide (AO/PI) staining. (**C**–**F**) The MFI of MHC I (H2-Kd), MHC II (IA/IE), CD80, and CD274 (PD-L1) was measured by flow cytometry. * *p* < 0.05, ** *p* < 0.01, and *** *p* < 0.001. All error bars are represented as mean ± standard deviation.

The current clinical goal after allo-HCT is to selectively reduce GvHD without compromising the GvL effect. Before evaluating the effect of BI-5756 on T cell-mediated anti-tumor activity, we assessed its direct anti-tumor effects independent of allogeneic T cells. A20, murine B cell lymphoma cells derived from BALB/c mice, were treated with BI-5756 for one day. BI-5756 suppressed A20 tumor cell growth in a dose-dependent manner without inducing cell death at the indicated concentrations below 20 μM (Figure 3A). At 40 μM, BI-5756 not only decreased A20 tumor cell numbers (Figure 3A) but also induced cell death in approximately 50% of A20 cells (Figure 3B). Interestingly, BI-5756 upregulated the expression of MHC I, MHC II, and the co-stimulatory molecule CD80 on A20 cells in a dose-dependent manner, indicating its potential as a promising anti-tumor agent that enhances T cell activation by increasing antigen presentation and co-stimulatory signaling on tumor cells (Figure 3C–E). Of note, BI-5756 did not affect the expression of CD274 (PD-L1), an immune checkpoint ligand that enables tumor immune evasion through the engagement of PD-1 on immune cells (Figure 3F). These results suggest that BI-5756 exerts direct anti-tumor activity without upregulating PD-L1 expression on tumor cells.

### 2.4. BI-5756 Does Not Compromise T Cell-Mediated Anti-Tumor Activities

**Figure 4:**
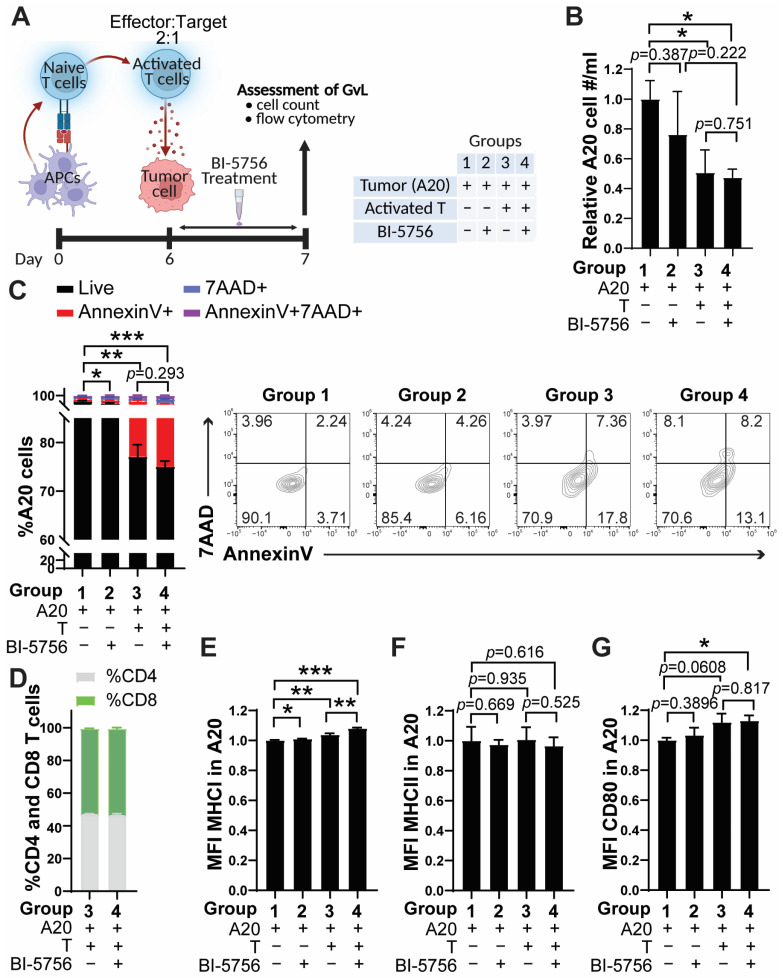
BI-5756 does not compromise T cell-mediated anti-tumor responses. (**A**) Schema of the co-culture experiment: MLR was performed using lethally irradiated (2000 rad) allogeneic APCs obtained from the spleens of BALB/c mice. After 6 days, only live pan T cells were isolated by Lympholyte-M cell separation media (Cedarlane Laboratories) and co-cultured with A20 tumor cells for 24 hrs in the presence or absence of 10 μM BI-5756. Experimental groups 1–4 are listed in the accompanying table. Created with BioRender.com (accessed on 27 August 2025). (**B**) The relative number (#) of A20 cells was calculated based on total viable cell counts (AO/PI staining), and the %A20 tumor cells was determined by flow cytometry. (**C**) The % cell death was assessed by Annexin V and/or 7AAD-positive cells (**left panel**), with representative flow cytometry plots shown (**right panel**). (**D**) The %CD4 and CD8 T cell ratio among total T cells (groups 3 and 4, right panel). The MFI of MHC I (**E**), MHC II (**F**), and CD80 (**G**) in A20 cells was analyzed by flow cytometry. * *p* < 0.05, ** *p* < 0.01, and *** *p* < 0.001. All error bars are represented as mean ± standard deviation.

To determine whether BI-5756 could maintain the beneficial anti-tumor activities of T cells, we performed a mixed lymphocyte reaction (MLR) using allogeneic APCs and B cell lymphoma A20 cells. Pan T cells from C57BL/6 mice were co-cultured with lethally irradiated (2000 rad) allogeneic APCs obtained from BALB/c mice for 6 days. After 6 days, only viable activated T cells were harvested by Lympholyte-M cell separation media and subsequently co-cultured with A20 cells for one day (Figure 4A). Remarkably, A20 cell growth was significantly suppressed in the presence of allogeneic T cells (Figure 4B). Although the treatment of BI-5756 did not further reduce A20 cell growth in the co-culture (Figure 4B), T cell-mediated anti-tumor response was preserved in the presence of BI-5756, as shown by the comparable tumor killing activity of T cells (Figure 4C). Consistent with Figure 1B, BI-5756 did not alter the ratio of CD4 and CD8 frequencies, even in co-culture with A20 and T cells (Figure 4D). Of note, BI-5756 slightly increased the expression levels of MHC I in A20 cells, and this upregulation was further enhanced in co-culture with allogeneic T cells (Figure 4E). In contrast, the expression levels of MHC II in A20 cells were not changed by either BI-5756 treatment or co-culture with allogeneic T cells (Figure 4F). Expression levels of CD80 in A20 were significantly increased in co-cultured cells treated with BI-5756 (Figure 4G). These results are consistent with a study from another group, which showed that ACEA, a CB1 agonist, also spares CD8 T cell cytotoxic activity [19], supporting the findings that BI-5756 preserves T cell-mediated anti-tumor activity.

### 2.5. CB1 Signaling Contributes to BI-5756-Mediated GvHD Reduction by Upregulating Tregs

**Figure 5:**
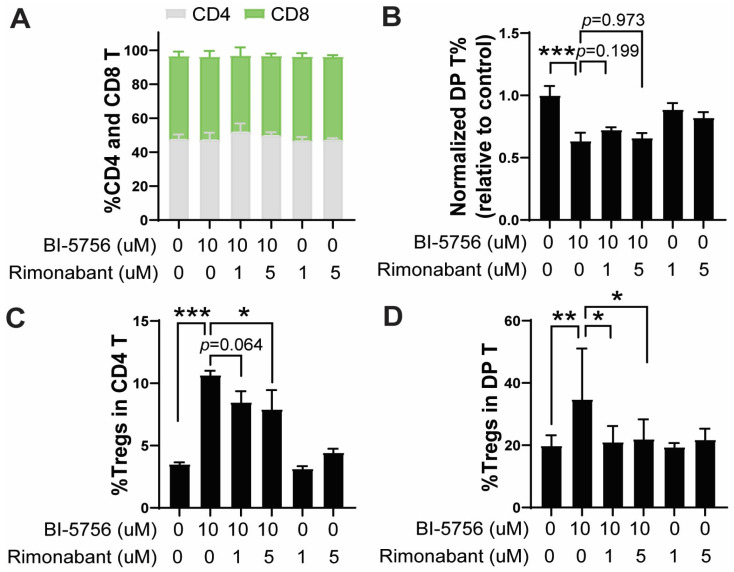
CB1 signaling contributes to BI-5756-mediated Treg upregulation. T cells were stimulated with anti-CD3/CD28 activation beads for 3 days in the presence or absence of BI-5756 (10 μM) and/or rimonabant, a CB1 antagonist, at the indicated concentrations. (**A**) The %CD4 and CD8, (**B**) normalized proportion of DP T cells, and (**C**) % Tregs were determined by flow cytometry. (**C**,**D**) Tregs were identified by GFP-positive cells within the CD4 or DP T cell population. * *p* < 0.05, ** *p* < 0.01, and *** *p* < 0.001. All error bars are represented as mean ± standard deviation.

Although BI-5756 is a CETP inhibitor, it demonstrates a potent CB1 agonist activity in both cell-based radioligand binding assay and functional GPCR GTP gamma assay. Since mice naturally lack CETP, we hypothesized that the immunomodulatory effects of BI-5756 are likely mediated through its primary off-target CB1. To test this hypothesis, we examined whether the pharmacological blockade of CB1 with rimonabant could abrogate BI-5756-mediated T cell immunomodulatory effects. The inhibition of CB1 using rimonabant did not change the ratio of CD4 and CD8 T cell frequencies (Figure 5A) and DP T cell proportions (Figure 5B), whereas it significantly reversed the BI-5756-induced increase in Treg frequencies in both CD4 (Figure 5C) and DP T cells (Figure 5D) stimulated with anti-CD3/CD8 activation beads. Of note is that rimonabant alone did not affect these T cell populations (Figure 5). These results strongly suggest that BI-5756 upregulates Tregs through the CB1 pathway in T cells, thereby reducing GvHD. Supporting our results, a previous study reported that rimonabant impaired the ability of human monocyte-derived tolerogenic DCs to prime allogeneic naïve T cells, indirectly reducing the generation of Tregs in a co-culture experiment.

## 3. Discussion

In this study, we identify BI-5756 as a novel and potent CB1 agonist with promising therapeutic potential for reducing GvHD in a preclinical mouse model of allo-HCT. In addition, we demonstrate that BI-5756 maintains T cell-mediated anti-tumor activity while increasing Treg frequencies in vitro. Although BI-5756 was originally developed as a CETP inhibitor, our data show that its effects on T cells are likely mediated through CB1 in primary murine T cells or mice after allo-HCT as rodents genetically lack CETP. BI-5756 exhibits a binding affinity to CB1 and effectively activates its downstream GPCR signaling. Our findings contribute to the emerging understanding of CB1 function in immune cells, particularly in T cells, which has been rarely explored compared to CB2, which is widely expressed in immune cells. We demonstrated that primary murine T cells express CB1 protein, even if it is at lower levels than CB2. This expression pattern of CB1 and CB2 in immune cells is consistent with the previous report on human PBMCs, in which mRNA transcripts of CB2 were more frequent than those of CB1 [27]. In addition, the expression of CB1 mRNA has been shown to be upregulated after T cell activation in response to CD3/CD28 in primary human T cells, supporting the expression of CB1 proteins in T cells, along with CB2 [28]. Functionally, BI-5756 significantly upregulated Tregs while suppressing the frequencies of CD4 and DP T cells, highly potent inducers of GvHD. Interestingly, BI-5756 spared CD8 T cell activation, as shown by selectively maintaining CD25 expression in CD8 T cells and their anti-tumor activity, which are critical for preserving GvL effects. The selective modulation of T cells by BI-5756 may reflect the characteristic of CB1 agonists such as ACEA, which have been shown to suppress T cell proliferation and activation without impairing the cytotoxic activities of CD8 T cells, unlike a CB2 agonist [11]. Thus, BI-5756 represents a promising CB1 agonist to reduce harmful GvHD without compromising the beneficial T cell-mediated anti-tumor response after allo-HCT. Indeed, consistent with its in vitro immunomodulatory effects, in vivo administration of BI-5756 significantly improved overall survival with less clinical GvHD in our preclinical mouse model of allo-HCT. In addition, recipient mice administered with BI-5756 showed a trend toward enhanced PLT count recovery and a significant increase in donor BM-derived B and T cell reconstitution, indicating positive outcomes after allo-HCT. The reduction in CD11b+Ly6C^high^ myeloid cells, which are considered to be inflammatory classical monocytes or neutrophils [29,30], can further support the anti-inflammatory capacity of BI-5756. Moreover, BI-5756 not only demonstrated direct anti-tumor activities but also maintained T cell-mediated anti-tumor response. All of these results suggest that the immunomodulatory effect of BI-5756 may be mediated by increasing Tregs while preserving T cell-mediated anti-tumor response by both direct and indirect mechanisms, including T cell-dependent and -independent tumor suppression and killing. The ability of BI-5756 to increase Tregs was significantly abrogated by the CB1 antagonist rimonabant. These findings are consistent with a previous report from another group showing that rimonabant impaired the ability of human monocyte-derived tolerogenic DCs induced by the synthetic cannabinoid WIN55212-2 to prime allogeneic naïve T cells, thereby reducing Treg generation [20]. Of note, CB1 has been shown to activate the mTOR pathway in various cell types, consistent with our observation of dose-dependent increases in mTOR activity upon BI-5756 treatment (Figure 1C). Although mTOR activation is typically associated with effector T cell differentiation, it can also support the homeostasis and functional activation of Treg by promoting IRF4 expression and reprogramming metabolism [31]. Therefore, it is conceivable that BI-5756-induced mTOR activation via CB1 signaling favors Treg expansion.

Consistent with a study on the CB1 agonist ACEA in T cells [11], BI-5756 also spares CD8 T cell activation and its anti-tumor activity. This may be explained by differential CB1 and CB2 expressions between T cell subsets, with CB2 being more abundantly expressed in CD8 T cells than CD4 T cells. Although we did not examine CB1 and CB2 protein expressions separately in CD4 and CD8 T cells, CB2 mRNA expression was more dominant in CD8 compared to CD4 T cells (Figure 1B). While CB1 mRNA expression was difficult to detect before activation (Figure 1B), we were able to detect both CB1 mRNA (Figure 1A) and protein expression (Figure 1C) in T cells after TCR stimulation by either allo-APCs (Figure 1A) or anti-CD3/CD28 activation beads. Therefore, it is conceivable that CB2 may be more functionally relevant in CD8 than CD4 T cells. This differential expression of CB1 and CB2 between CD4 and CD8 T cells could explain why CB2 fails to spare CD8 T cell function, in contrast to CB1 agonists. Thus, selectively targeting CB1 while sparing CB2 may offer a novel strategy to reduce GvHD while maintaining the GvL effect after allo-HCT.

BI-5756 is expected to provide clinical benefits in humans through CETP inhibition, as CETP inhibition is known to increase plasma levels of high-density lipoprotein HDL-C while decreasing those of low-density lipoprotein LDL-C [32,33]. Elevated plasma levels of HDL-C have been reported to enhance Treg development and function and exert anti-inflammatory effects by inhibiting dendritic cell (DC) activation and inflammatory cytokine production [34,35,36]. Of note, the administration of HDL to mice undergoing allo-HCT significantly improved overall survival and reduced GvHD severity by lowering circulating endotoxin, well-known GvHD triggers and amplifiers from intestinal gram-negative bacteria, and systemic (serum IL-6) and local inflammation (Kupffer cell-derived IL-12) [34]. Moreover, HDL-deficient (Apoa1^tm1unc^) recipient mice developed exacerbated GvHD. Since rodents lack the CETP gene, we could investigate BI-5756’s potential main off-target effect as a CB1 agonist in our mouse model of allo-HCT. Based on our current study, BI-5756 may exert additive/synergistic effects in reducing GvHD in patients after allo-HCT by concurrently inhibiting CETP and activating CB1. Clinical studies have also reported an association between hypercholesterolemia in either recipients or donors at the time of transplantation and an increased GvHD incidence, although statistical significance was limited by a small sample size [37]. As studies on CBs in T cells and allo-HCT remain largely unexplored, further investigation and validation using diverse CB1 and CB2 agonists and antagonists are warranted. Moreover, even though BI-5756’s properties as a CETP inhibitor and CB1 agonist may enhance its translational relevance, its potential psychoactive effects, including mood changes, altered memory, and cognitive impairment, as observed with endogenous and exogenous cannabinoids such as THC, along with other side effects, particularly those that are time- and dose-dependent in long-term use, must be carefully monitored and investigated.

In conclusion, our findings suggest that BI-5756 provides new insights into the role of CB1 in GvHD after allo-HCT. A major clinical goal after allo-HCT is to reduce GvHD while maintaining the GvL effect, as standard immunosuppressive strategies often reduce GvHD but also impair beneficial anti-tumor responses, given that donor T cells mediate both GvHD and GvL. Importantly, our study suggests that BI-5756 may help overcome this challenge, as it reduces GvHD in our mouse model of allo-HCT while preserving T cell-mediated anti-tumor activity in vitro. Due to its unique therapeutic potential to selectively modulate T cell responses in GvHD but not in anti-leukemia effects, as well as lipid-cholesterol metabolism by regulating both CETP and CB1, BI-5756 will also provide broader implications beyond allo-HCT, including solid organ transplant rejection and other inflammatory diseases.

## 4. Materials and Methods

### 4.1. Compounds

BI-5756 was kindly provided by Boehringer Ingelheim via its open innovation platform opnMe, available at https://www.opnme.com. The opnMe^®^ open science portal offers comprehensive information on BI-5756, including its chemical structure, target specificity and selectivity, X-ray crystal structure, in vitro activity data, reference molecules, in vitro and in vivo drug metabolism and pharmacokinetics (DMPK) parameters, and in vitro chemistry, manufacturing, and control (CMC) data. Stock solutions were prepared by dissolving BI-5756 in 100% DMSO, aliquoted, and stored at −80 °C. For in vitro studies, DMSO was used at <0.1% as the corresponding vehicle control. For in vivo administration, DMSO stocks were thawed and diluted with 0.5% hydroxyethyl cellulose. In addition, 10% DMSO and 0.45% hydroxyethyl cellulose were used as the corresponding vehicle control.

Rimonabant (#HY-14137) and CB1 agonist 1 (#HY-148137) were obtained from MedChemExpress (Monmouth Junction, NJ, USA). Stock solutions were prepared in 100% DMSO, aliquoted, and stored at −80 °C. DMSO was used at <0.1% as the corresponding vehicle control.

### 4.2. Mice

All male C57BL/6 (H2-Kb, CD45.1, or CD45.2) and BALB/c (H2-Kd and CD45.2) mice were purchased from the Jackson Laboratory (Bar Harbor, ME, USA). Foxp3 and EGFP co-expressing (B6.Cg-Foxp3tm2Tch/J) mice were also obtained from the Jackson Laboratory. All animals were housed under specific pathogen-free conditions. Mice were 7–12 weeks of age at the start of the experiments. All animal protocols were approved by the Institutional Animal Care and Use Committee (IACUC) at Washington University in St. Louis, MO, USA.

### 4.3. RNA Sequencing

Mouse primary splenocytes were isolated from spleens, and resting CD4 and CD8 T cells (CD25-) were purified using a Sony Synergy cell sorter (Sony Biotechnology Inc., San Jose, CA, USA). Total RNA was extracted using the RNeasy Universal Kit (Qiagen, Germantown, MD, USA), and RNA library preparation and sequencing were performed by Novogene Corporation Inc. (Sacramento, CA, USA) following the WBI-Quantification reference. The prepared libraries were sequenced on the NovaSeq platform (Illumina, San Diego, CA, USA). In addition, RNA profiling data from activated CD4 T cells in our previous report were retrospectively analyzed and generated to assess CB1 and CB2 expressions [38].

### 4.4. Real-Time Quantitative PCR (qPCR)

cDNA was synthesized from 1 µg of total RNA using Moloney murine leukemia virus (M-MLV) reverse-transcriptase and oligo (dT) primer (Promega, Madison, WI, USA) and subsequently used for qPCR. qPCR was performed with SYBR Green qPCR Master Mix (2×; USB products; Affymetrix, Santa Clara, CA, USA) on a QuantStudio™ 3 Real-Time PCR System (Applied Biosystems, Carlsbad, CA, USA). The PCR primer pairs used are as follows: *Ifng*: 5′-ATGAACGCTACACACTGCATC-3′, 5′-CCATCCTTTTGCCAGTTCCTC-3′; *Il1a*: 5′-CGAAGACTACAGTTCTGCCATT-3′, 5′-GACGTTTCAGAGGTTCTCAGAG-3′; *Il6*: 5′-TAGTCCTTCCTACCCCAATTTCC-3′, 5′-TTGGTCCTTAGCCACTCCTTC-3′, *Il10*: 5′-GCTCTTACTGACTGGCATGAG-3′, 5′-CGCAGCTCTAGGAGCATGTG-3′, *Tgfb*: 5′-CTCCCGTGGCTTCTAGTGC-3′, 5′-GCCTTAGTTTGGACAGGATCTG-3′, *Il4*: 5′-GGTCTCAACCCCCAGCTAGT-3′, 5′-GCCGATGATCTCTCTCAAGTGAT-3′, *Tnfa*: 5′-CCCTCACACTCAGATCATCTTCT-3′, and 5′-GCTACGACGTGGGCTACAG-3′.

Mouse *Gapdh* primers were purchased from Qiagen (Cat#QT01658692, Hilden, Germany).

### 4.5. Primary Murine T Cell Culture

Spleens were harvested from mice and dissociated using the back end of a 1 mL syringe plunger to generate single cell suspensions. The single suspensions were passed through a 70 μM mesh nylon strainer. Subsequent Pan T cell isolation was performed on the single cell suspension using the EasySep™ Mouse T Cell Isolation Kit according to the manufacturer’s instructions (STEMCELL, #19851, Vancouver, BC, Canada). Isolated T cells were checked for their purity by flow cytometry and cultured in Xcyte media supplemented with 10% fetal bovine serum (FBS), 1% penicillin–streptomycin, 2 mM GlutaMAX, 12.5 mM HEPES, 10 μg/mL ciprofloxacin, 50 μM β-mercaptoethanol, and 10 IU/mL recombinant human IL-2 in a DMEM base. β-mercaptoethanol and IL-2 were added immediately before use. Pan T cells were seeded at 0.5 × 10^6^ cells/mL and activated with Dynabeads Mouse T-activator CD3/CD28 (Thermo Fisher Scientific, #11453D, Waltham, MA, USA) at a 1:1 bead-to-cell ratio. Cultures were maintained at 37 °C in a humidified incubator with 5% CO_2_ for 2–3 days.

### 4.6. Preclinical Mouse Model of Allo-HCT

Allo-HCT (C57BL/6 to BALB/c) was performed as previously described [8,38,39,40,41,42]. In the GvHD model, 5 × 10^6^ T cell-depleted bone marrow cells (TCD-BMs) from CD45.1+ C57BL/6 (H-2b) were injected at day 0 after the lethal irradiation (900 cGy at day^−1^) of CD45.2+ BALB/c (H-2d) recipient mice. Eleven days after, delayed lymphocyte infusion (DLI) was performed using 2 × 10^6^ splenic pan T cells from CD45.2+ C57BL/6 mice. Mouse TCD-BM cells were prepared using EasySep mouse CD90.2 positive selection kit II (STEMCELL Technologies, Cambridge, MA, USA). Mouse pan T cells (CD4+ and CD8+ T cells) were isolated from mouse spleens using an EasySep mouse T cell isolation kit (STEMCELL Technologies, Cambridge, MA, USA). Mice receiving TCD-BMs only served as GvHD-negative controls. All mice were monitored and weighed once or twice per week for survival and signs of GvHD for up to 60 days, as we previously reported [38,39,42]. Surviving mice were bled on day 28 for complete blood counts and BM reconstitution analysis by flow cytometry. Clinical GvHD scoring was assessed based on 5 categories: weight loss, posture, activity, fur texture, and skin integrity, with a maximum of 2 points per category [43].

### 4.7. Western Blotting

Primary murine T cells were harvested on day 3 after activation with Dynabeads Mouse T-activator CD3/CD28 and lysed in M-PER™ Mammalian Protein Extraction Reagent (Thermo Fisher Scientific, #78503, Waltham, MA, USA) supplemented with protease and phosphatase inhibitors and DNase I. Total protein concentrations were determined using the BCA protein assay. Equal amounts of protein (20–50 μg per lane) were separated by SDS-PAGE at 100 V until the dye front reached the bottom of the gel, followed by transfer to PVDF membranes at 80 V for 2 h. Membranes were blocked in 5% non-fat dry milk in PBS-T (PBS with 0.1% Tween 20) at room temperature (RT) for 20 min to reduce non-specific binding. After blocking, membranes were incubated with primary antibodies for CB1 (Cell Signaling, #93815, Danvers, MA, USA), CB2 (Invitrogen, #703485, Waltham, MA, USA), phosphor-S6 ribosomal protein (Cell Signaling, #4856), or α-tubulin (Cell Signaling, #2144) (one at a time). After each primary antibody incubation, membranes were washed with PBS-T and incubated with anti-rabbit HRP-conjugated secondary antibody for 30 min at RT. Following 3 washes with PBS-T, protein bands were visualized using the ChemiDoc™ MP Imaging System (Bio-Rad, #12003154, Hercules, CA, USA).

### 4.8. Flow Cytometry Analysis

Primary murine T cells were harvested on day 2 or day 3 after anti-CD3/28 bead activation or on day 6 after MLR and transferred into FACS tubes. Cells were washed with FACS buffer containing 0.5% bovine serum albumin (BSA) and 2 mM Ethylenediaminetetraacetic acid (EDTA). Following the wash, cells were incubated with 2% rat serum at RT for 5 min. For staining, cells were incubated with a master mix of fluorescent-conjugated antibodies at 4 °C for 30 min in the dark (wrapped in foil). BD Horizon™ Brilliant Stain Buffer (BD Biosciences, #563794) was used if the fluorescent master mix contained more than one Brilliant Violet fluorophore. The antibodies used for mouse cells were purchased from BioLegend (San Diego, CA, USA) and BD Biosciences (Franklin Lakes, NJ, USA), as follows: from BioLegend: CD3 (#100236), H2-Kb (#116520), H2-Kd (#116608), CD274 (#124314), and Ly6C (#128028); from BD Biosciences, CD4 (#563050), CD25 (#557658), B220 (#563893), CD11b (#561039), IA/IE (#742894), CD8 (#563068), Annexin V (#556422), and CD80 (#560523). To determine FOXP3-positive Tregs, we utilized Foxp3 and EGFP co-expressing C57BL/6 mice as T cell donors, allowing for direct detection of GFP-positive Tregs without intracellular staining. After staining, cells were washed with FACS buffer and analyzed using an Attune NxT Flow Cytometer (Thermo Fisher Scientific, Waltham, MA, USA).

### 4.9. Statistical Analysis

All data are presented as mean ± standard deviation (SD). Statistical analyses were performed using GraphPad Prism version 10.5.0 (GraphPad Software, San Diego, CA, USA). Survival data were analyzed using the log-rank test. For all comparisons between groups, unpaired two-tailed Student’s *t*-tests and one-way or two-way analysis of variance (ANOVA) were used. In addition, *p*-values less than 0.05 were considered statistically significant. The number of biological replicates is indicated in the figure or figure legends. All experiments were repeated at least twice to ensure reproducibility of results.

## Data Availability

The data presented in this study are contained within the article and are also available upon request from the corresponding author.

## Supplementary Materials

The following supporting information can be downloaded at: https://www.mdpi.com/article/10.3390/molecules30173517/s1, Figure S1. Cytokine expression at the mRNA level in the absence or presence of BI-5756. Figure S2. CB1 agonist 1 also upregulates regulatory T cells in primary murine pan T cell cultures.
