# Supplementary material for: BI-5756 Reduces Graft-Versus-Host Disease Through CB1-Mediated Treg Upregulation
Source: Molecules. 2025 Aug 28;30(17):3517. doi: 10.3390/molecules30173517 (PMC12430402; doi:10.3390/molecules30173517)
Supplement: Supplementary file 1 [file molecules-30-03517-s001.zip › molecules-3755280-supplementary.pdf]

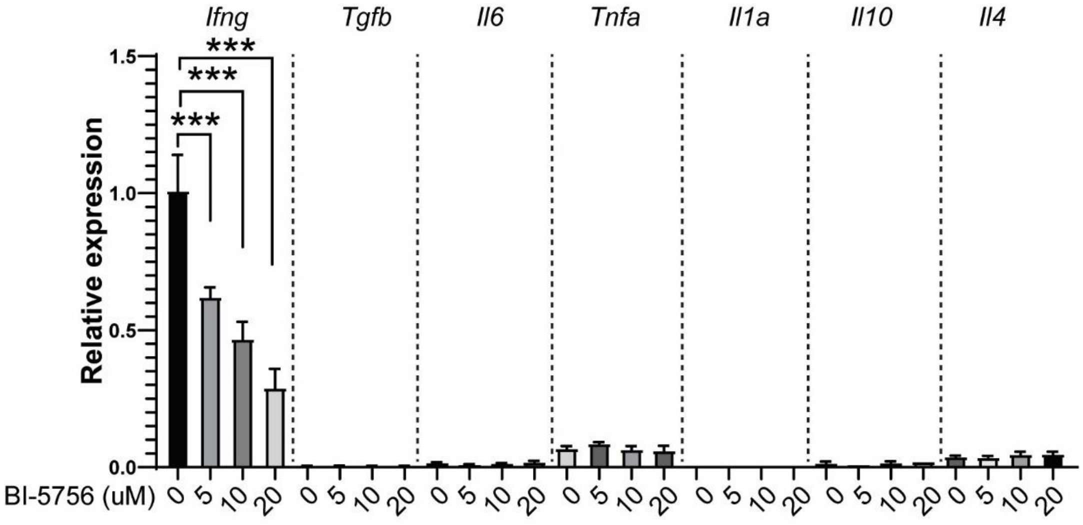

Supplementary Figure 1. Cytokine expression at the mRNA level in the absence or presence of BI-5756. T cells were stimulated with anti-CD3/CD28 activation beads for overnight, and cytokine expressions were determined by quantitative PCR. Relative mRNA expressions were calculated using the ΔΔCt method. ***p <0.001. All error bars are represented as mean ± standard deviation.

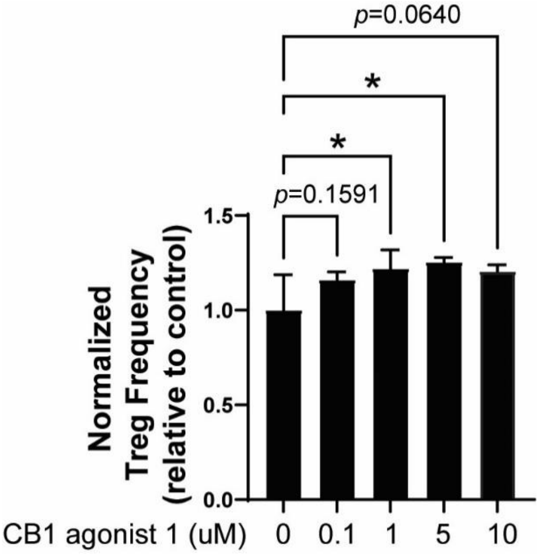

Supplementary Figure 2. CB1 agonist 1 also upregulates regulatory T cells in primary murine pan T cell cultures. T cells were stimulated with anti-CD3/CD28 activation beads for 3 days in the presence or absence of CB1 agonist 1. %Tregs were determined by flow cytometry and normalized to the control. Tregs were identified by GFP-positive cells within the CD4 T cell population. *p<0.05. All error bars are represented as mean ± standard deviation
